# Incidental cystadenocarcinoma of the appendix in a patient undergoing laparoscopic right hemicolectomy for caecal cancer: a case report

**DOI:** 10.4076/1757-1626-2-7505

**Published:** 2009-06-09

**Authors:** Caroline LE Osborne, Caroline E Boulind, Edwin Cooper, Nader K Francis

**Affiliations:** Department of Surgery, Yeovil District Hospital Foundation TrustHigher Kingston, Yeovil, SomersetUK

## Abstract

Primary appendiceal neoplasms are rare and usually found incidentally after appendicectomy for suspected appendicitis. We report a case of a perforated cystadenocarcinoma of the appendix occurring synchronously with caecal adenocarcinoma in an 81-year-old woman without abdominal symptoms or signs, who presented with iron deficiency anaemia.

## Introduction

Primary appendiceal neoplasms are uncommon [1] and usually found incidentally after appendicectomy for suspected appendicitis [2]. Preoperative diagnosis is frequently difficult. Whilst the symptoms are typically non-specific, imaging studies are not usually diagnostic [3]. Here, we report a case of cystadenocarcinoma of the appendix presenting asymptomatically and occurring synchronously with caecal adenocarcinoma. This rare but important incidental finding in a patient without any abdominal signs or symptoms is significant for the management and prognosis of the patient. A laparoscopic approach allowed excellent assessment of the disease and its extent without compromising oncological principles or further therapeutic intervention that may be required.

## Case presentation

**Figure 1.**
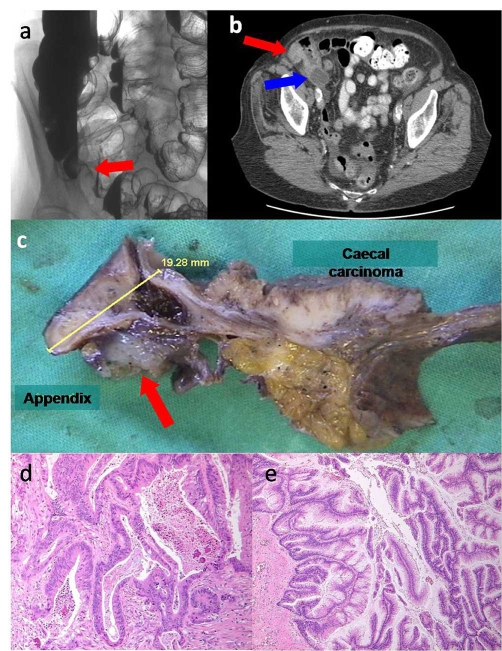
Imaging of the colon demonstrated indentation of the caecal wall (**A**, red arrow) on air contrast barium enema and thickening of the caecal pole (**B**, red arrow) and a mucocele of the appendix (**B**, blue arrow) on CT scan with oral contrast. Examination of the gross specimen **(C)** showed a flat tumour of the caecum and a separate lesion in the appendix. The appendix had ruptured and mucinous deposits had extruded onto the serosa (**C** red arrow). Haematoxylin and eosin stained sections of the two tumours demonstrated distinct morphologies. The caecal tumour showed complex glandular structures lined by highly atypical cells infiltrating through the bowel wall and inciting an inflammatory and stromal desmoplastic reaction indicative of invasive adenocarcinoma **(D)**. By contrast, the appendiceal lesion showed an architecture resembling adenoma, composed of villous structures lined by columnar cells with mucin vacuoles **(E)**. The basally located nuclei showed only mild cytological atypia and the proliferative activity as measured by immunostaining for Ki-67 was predominantly at the basal layer. The lesion appeared to have an expansile growth pattern without evidence of destructive infiltration of the wall of the appendix. Although mucin was seen on the outer serosal surface, no viable tumour cells were identified outside the lumen of the appendix.

An 81-year-old Caucasian woman was admitted with a two week history of increasing shortness of breath and dizziness. Although frail she was otherwise medically fit, a non-smoker and had no significant family history. She had a history of endometrial cancer, treated 14 years previously with hysterectomy, bilateral salpingo-oopherectomy and adjuvant radiotherapy. The onset of her symptoms was gradual and comprised malaise, shortness of breath initially on exertion and more lately at rest and dizziness especially on standing up from sitting. She denied any symptoms related to her gastrointestinal tract such as haematemesis, melaena, change in bowel habit or abdominal pain. On examination she appeared pale and was dyspnoeic, tachycardic and normotensive. Examination of the abdomen revealed it to be soft and non-tender, with no palpable abdominal masses. Blood tests showed a microcytic, normochromic anaemia with a haemoglobin of 3.8 gm/dL and a carcinoembryonic antigen level of 15 ng/ml. Blood biochemistry was within normal limits. After blood transfusion sources of intestinal haemorrhage were sought; an upper gastrointestinal endoscopy was unremarkable and colonoscopy was unhelpful because a fixed rectum, secondary to previous surgery and radiotherapy for endometrial cancer impeded the progress of the scope. An air contrast barium enema examination was performed and this showed an indentation of the caecal wall (Figure 1a) suggestive of a caecal tumour. A computerized tomography scan demonstrated thickening of the caecal pole and a mucocele of the appendix (Figure 1b) suggesting a caecal tumour that causing obstruction of the appendiceal orifice. There was no sign of extracolonic or metastatic disease. After counselling the patient and her family, it was decided the best treatment option was to perform a laparoscopically assisted right hemicolectomy. At laparoscopy, the diagnosis of a mucolcele of appendix was confirmed. The appendiceal mucocele was perforated and surrounded by mucinous deposits. The caecum appeared bulky and abnormal. A laparoscopic assisted right hemicolectomy and partial omentectomy were performed without complication. Postoperatively the patient developed hyponatraemia and a paralytic ileus, delaying her recovery. She was discharged eight days after the surgery and followed up in the outpatients two weeks later when she reported feeling well recovered from the surgery.

Histology of the resected specimen demonstrated a T3N1M0 adenocarcinoma of the caecum with 2 out of 14 lymph nodes containing tumour cells. Surprisingly a mucinous neoplasm of the appendix was also identified, separated from the caecal tumour by a segment of normal caecal mucosa. Each tumour had a distinct morphology. The cytological characteristics of the caecal tumour were typical of a moderately differentiated colonic adenocarcinoma (Figure 1d), whist the appendiceal tumour had a striking viliform structure with abundant basal nuclei and mucin extruding through and onto the serosal surface (Figure 1e). Neoplastic cells were not seen on the appendix serosa or within the mucinous deposits. Immunohistochemistry using an antibody against p53 (not shown) demonstrated over expression of the abnormal protein in the caecal lesion, whereas the appendiceal tumour was devoid of positive staining. There were also differences in cell proliferation rates in the two tumours, as demonstrated by immunostaining with Ki67 (not shown), which was strongly positive in the caecal carcinoma but weak in the appendiceal lesion. No abnormal cells or mucin were detected in the resected omentum.

## Discussion

Neoplasia of the appendix, although rare, is an important diagnosis to consider in patients with a mucocele of the appendix. The clinical presentation of tumours of the appendix can be similar to acute appendicitis and frequently the diagnosis is made incidentally [2]. The frequency of appendix specimens removed for suspected appendicitis that contain a neoplasm is 0.08% [4]. The diagnosis of cystadenocarcinoma of the appendix in this case was surprising as we had assumed that the mucocele was secondary to obliteration of the appendiceal orifice by caecal cancer. Pre-operative diagnosis is often challenging and infrequent but when a mucocele of the appendix is identified, a primary appendiceal tumour should be considered. CT imaging typically identifies a cystic nodular mass with an enhancing wall in the right iliac fossa [5]. Perforation of the mucocele resulting in dissemination of tumour cells can occur and examination of the peritoneal cavity in our patient revealed mucinous deposits adjacent to the perforated appendix. Interestingly, when a patient presents with a cystadenocarcinoma of the appendix a second gastrointestinal tumour is present in 20-40% of cases [4]. The synchronous caecal tumour in our patient necessitated full resection of the right hemicolon and its mesentery, which was performed laparoscopically and with adherence to oncological principles. Evidence supporting a laparoscopic approach to treatment for cystadenocarcinoma of the appendix is limited to a few reports, although resection of other primary appendiceal tumours has been safely effected using laparoscopic surgery [6]. In our experience, performing the resection laparoscopically did not compromise oncological safety and in fact we were afforded excellent views of the whole peritoneal cavity allowing assessment of disease extent and good access to remove all visible mucinous deposits.

## Conclusion

Cystadenocarcinoma of the appendix is an important diagnosis to consider in patients diagnosed with a mucocele of the appendix. Although the these tumours usually present clinically like acute appendicitis, this case was interesting because the mucocele remained completely asymptomatic despite perforation and the patient presented with systemic symptoms related to a second colonic neoplasm.
